# Beyond accommodation: on the structural turn in computational functionalist theories of consciousness

**DOI:** 10.1093/nc/niaf014

**Published:** 2025-06-06

**Authors:** Francesco Ellia, Naotsugu Tsuchiya

**Affiliations:** Laboratory of Qualia Structure, ATR Computational Neuroscience Laboratories, 2-2-2 Hikaridai, Seika-cho, Soraku-gun, Kyoto 619-0288, Japan; School of Psychological Sciences, Monash University, Melbourne, Victoria, Australia; Laboratory of Qualia Structure, ATR Computational Neuroscience Laboratories, 2-2-2 Hikaridai, Seika-cho, Soraku-gun, Kyoto 619-0288, Japan; School of Psychological Sciences, Monash University, Melbourne, Victoria, Australia; Center for Information and Neural Networks (CiNet), National Institute of Information and Communications Technology (NICT), Suita-shi, Osaka 565-0871, Japan

**Keywords:** Consciousness, Theories and models, Computational Functionalism, Functionalism, Structure, Integrated Information Theory

## Abstract

This commentary engages with recent work on computational functionalist theories of consciousness through a structural lens. We address three key aspects: the role of subjective experience in theory building, the hypothesis regarding local lateral connectivity in sensory areas, and the implications of “silent units” for consciousness. We argue that while their structural turn is welcome, many of their insights were previously predicted by Integrated Information Theory. We question the coherence of these claims within the functionalist paradigm and emphasize the importance of distinguishing genuine predictions from post-hoc accommodations in consciousness science.

## Introduction

In a recent article, Fleming and Shea (2024) offer a new perspective on computational functionalist theories of consciousness, refocusing them through the lens of a structural approach. By the author’s recognition, traditionally, computational functionalist approaches have mostly ignored the structural properties of experience. On the contrary, structural properties of subjective experience have long been emphasized by other theories, among which most prominently is Integrated Information Theory (IIT, Albantakis et al. 2023, Hendren et al. 2024, Tononi et al. 2025). Here, functionalism refers to theories that explain consciousness in terms of what a system does (e.g. its computational or information-processing functions). In contrast, structuralist theories employ mathematical structures to account for the structure of subjective experience. In this commentary, we engage with the three issues presented in Fleming and Shea’s work: the constraints imposed by subjective experience in theory building, the hypothesis regarding local lateral connectivity in the sensory areas as a substrate for subjective experience, and the role of “silent units” for consciousness while employing a structural stance. Finally, we offer some meta-theoretical reflections on the interplay between evidence and theory that we deem crucial for future progress in the field.

## Subjective experience and its constraints in theory building

Fleming and Shea write: “Hence, perhaps surprisingly, the nature of phenomenal character furnishes evidence for and against some theories of consciousness” (Fleming and Shea 2024). Peters (Peters 2025) has also recently advocated for the importance of introspection within the functionalist framework. However, the idea that subjective experience provides crucial constraints on how a theory is developed is neither new (Tononi 2004) nor surprising. A scientific theory of consciousness should account for subjective experience objectively (Ellia et al. 2021). Thus, if phenomenal experience (its presence or absence, and its qualitative character) is the explanatory target of any aspirational theory of consciousness, we find it trivially true that “the nature of phenomenal character” must play a central role in constraining theory-building itself and arbitrating between competing frameworks. Indeed, recently popularized no-report paradigms (Tsuchiya et al. 2015) strongly indicate that the field has taken phenomenology seriously.

Moreover, as Song (2024) remarks in reply to Fleming and Shea, explaining the qualitative aspect of consciousness—its phenomenology—is a daunting task. While quality spaces can provide formal descriptions of phenomenal relationships, we argue that simply mapping mental states onto such spaces from an external viewpoint does not fully explain why particular experiences have the specific qualitative character they have (Lee-Youngzie et al. 2024, Tsuchiya 2024). This remains true even when such spaces are grounded in neural dynamics, as the fundamental question of *why* certain neural patterns correspond to particular experiential qualities persists (Chalmers 1995).

Unless functionalist theoreticians propose a mechanism that accounts for the qualitative character of experience from the system’s intrinsic perspective, this framework remains insufficient (Gomez-Marin 2023). Merely mapping mental states onto an arbitrary quality space from an external viewpoint does not explain *why* particular experiences have the qualitative feel they do to the subject. In contrast, Integrated Information Theory attempts to explain consciousness from first principle by considering how the system’s internal organization and dynamics can account for its phenomenology. Without a mechanism that intrinsically bridges functional states to subjective qualities, computational functionalism may fail to fully address the fundamental question of why experiences feel a certain way to the experiencing system itself, a problem already noticed by Chalmers (1995).

## On the role of local lateral connectivity

Discussing Malach’s proposal (Malach 2021), the authors engage with the hypothesis of lateral cortical connectivity as a neural substrate for relational geometry, quoting from Fleming and Shea (2024):

“According to this account, local lateral connections in sensory brain areas implement a quality space for each sensory domain. Since representational geometry is defined by the neural activation patterns triggered by inputs, the brain network responsible will also depend on the strength of inputs to an area and on feedback connections. In turn, lateral connectivity itself (and, therefore, the quality space) will be modulated by top-down neuromodulatory connections.” (Fleming and Shea 2024).

As noted by the authors, this hypothesis finds empirical relevance, thanks to the works of Song, Haun, and Tononi (Song et al. 2017):

“Some intriguing psychophysical evidence for this view was obtained by Song et al. […] These findings suggest that a local change *in the strength of lateral connections is sufficient to alter experience of visuospatial relations*.” (Fleming and Shea 2024, emphasis added).

While this hypothesis might appear novel to computational functionalist approaches, it has been a critical prediction of IIT for over two decades, as discussed by Tononi:

“In summary, these simulations indicate that, even if the anatomical connectivity of a complex stays the same, a change in key parameters governing the readiness of neurons to respond and the differentiation of their responses may alter radically the Φ value of the complex, with corresponding consequences on consciousness”. (Tononi 2004)

“IIT also predicts that the spatial structure that characterizes much of our daily experience should be reflected in features of conceptual structures that are specified by connections among neurons arranged in two-dimensional grids *[…] This also implies that local strengthening or weakening of such horizontal connections in topographic areas should lead to a local distortion of experienced visual space*, even though the feedforward mapping of visual inputs from the world remains unchanged”. (Tononi et al. 2016, emphasis added)

Why do structural, but not functional, theories of consciousness make such claims? Haun and Tononi (2019) proposed a principled explanation of why space feels the way it does. In their proposal, they identify the essential characteristics of the phenomenology of space as reflectivity, connection, fusion, and inclusion. Such properties are accounted through an explanation in cause-and-effect terms; it follows directly from this theoretical account that changes in local connectivity have severe implications for the experience of space. This point is strictly connected with another long-time-standing prediction of IIT regarding inactive and inactivated units:

“It should also be emphasized that, in every case, it is the activity state of *all* elements of the complex that defines a given conscious state, and both active and inactive elements count.” (Tononi 2004)

“Take elements (neurons) within the main complex that happen to be silent when one is having a particular experience. If one were to temporarily disable these neurons (e.g., make them incapable of firing), the prediction is that, though the system state (firing pattern) would remain the same, the quantity and quality of experience would change (Balduzzi & Tononi 2004, 2008)”. (Tononi 2008)

This prediction is currently being tested in an adversarial collaboration (Olcese et al. 2024). Taking IIT at face value, it follows from its principled account that the strength of connectivity and the inactive or inactivated state of relevant units play a central role in determining both the presence and the qualitative aspects of consciousness. This suggests that, even if the exact formalization of IIT’s postulates were to change, the fundamental claim about the importance of connectivity would remain the same.

In contrast, functionalist theories focus on computational processes and functional organization (Doerig et al. 2019). It is therefore not immediately clear why functionalist accounts, such as those mentioned by Fleming and Shea (2024), make a direct connection between lateral connectivity and the implementation of a quality space. Unlike IIT, where the relevance of lateral connectivity and inactive units emerges from foundational axioms, functionalist theories presented here have not yet provided a principled explanation for why such structural features should impact conscious experience. While this does not preclude the possibility of functionalist theories developing such explanations, or suggest that explanations must necessarily derive from first principles, we believe that a satisfactory account has not yet been presented. Therefore, this raises questions about the coherence of these claims within the functionalist paradigm. For us, this discrepancy serves as a test case for determining whether a given theory is fundamentally structuralist or functionalist in nature (Ellia & Tsuchiya, manuscript in preparation; Tsuchiya et al. 2020).

## From phenomenology to functions, not from functions to phenomenology

For IIT, phenomenology constitutes the primary explanandum that can and should be explained from a structuralist perspective. This is clear from IIT’s stress in starting from phenomenological properties (i.e. intrinsicality, information, integration, exclusion, and composition), which implies that any experience has structural properties. IIT then postulates the physical properties that are necessary to implement these phenomenological properties, which lead to an explanation of the structure of phenomenal experience in terms of the cause–effect structure of a physical system.

In summary, for IIT, the flow of explanation moves from phenomenological structure to the cause–effect structure of the physical substrate of consciousness.

When applied to evolutionary contexts, this explanation can elucidate potential adaptive advantages for organisms to have consciousness, such as a better “matching” between the structure of the organism’s experience and the complexity of its environment (Mayner et al. 2024). Note, however, that IIT also predicts that under some contexts, not having consciousness can be more advantageous. Specifically, when the environment is highly regular and simple, the most evolutionary adaptive animals can be feed-forward systems, which IIT predicts to be nonconscious (Albantakis et al. 2014).

Functionalist theories approach consciousness through its functional relationships, although they do so in diverse ways. Some examine what functions give rise to consciousness, while others investigate what functions consciousness enables. These approaches differ in whether they identify functions necessary for consciousness, sufficient for it, or both. What unites functionalist accounts is their methodological emphasis on function rather than any specific claim about particular functions. For example, while some functionalists argue that consciousness integrates information across cognitive modules, others focus on its role in enabling flexible decision-making or facilitating learning. These theories vary significantly in how they conceptualize the relationship between consciousness and its functions, whether consciousness can be fully reduced to functional roles, and whether multiple functional pathways can realize the same conscious states (Niikawa et al. 2022).

While functionalist insights about consciousness’s adaptive roles deserve serious consideration, we argue that the reverse flow of inference—from functions to phenomenological structures—faces fundamental challenges. As Chalmers (1995) argues, even if we can fully describe the functional architecture supporting conscious behavior, we still cannot bridge the explanatory gap between functional organization and subjective experience.

## Predictions and accommodations

Distinguishing genuine predictions from accommodations is essential to the advancement of science. In his theory-appraisal framework for consciousness science, Negro (2024) posits that it is acceptable for theories to “predict” known facts, provided these facts have not been anticipated by competing research programs. However, as traditionally emphasized in the philosophy of science literature (Barnes 2022), a theory cannot claim to predict known facts that have already been predicted by rival theories; such instances are mere accommodations of these facts. Therefore, within this framework, the predictive and explanatory power of the theoretical approaches discussed by Fleming and Shea—including Fleming’s own Higher-Order State Space (Fleming 2020)—is significantly diminished.

We have highlighted how Fleming and Shea have aligned computational functionalist theories with a broader structural turn within consciousness science (Kleiner 2024). While this is a highly welcomed development, it also invites questions about the coherence of these claims within the functionalist paradigm and suggests a possible convergence toward structural considerations traditionally associated with IIT.

We believe that even greater progress might be achieved by fully embracing the intrinsic perspective, and if genuinely distinguishing predictions are generated based on a deeper understanding of the currently proposed theories. We therefore invite proponents of computational functionalist theories to clearly distinguish between novel facts predicted by their theories and known facts that are merely accommodated.

## Data Availability

No new data were generated or analyzed in support of this research.
